# From Assessment to Action: A Research Prototype for SIPAT-Based Multidomain Psychosocial Visualization and Prioritization in Lung Transplant Candidates

**DOI:** 10.3390/jcm15155899

**Published:** 2026-07-28

**Authors:** Aleksandra Stańska, Wojciech Karolak, Jacek Wojarski

**Affiliations:** 1 Division of Quality of Life Research, Department of Psychology, Faculty of Health Sciences, Medical University of Gdańsk, 80-210 Gdańsk, Poland; 2 Department of Cardiac & Vascular Surgery, Faculty of Medicine, Medical University of Gdańsk, 80-210 Gdańsk, Poland; wkarolak@gumed.edu.pl (W.K.);

**Keywords:** lung transplantation, SIPAT, psychosocial assessment, clinical decision support, machine learning, random forest, psychosocial risk, prehabilitation, treatment adherence, risk stratification

## Abstract

**Background:** Psychosocial assessment is a core component of lung transplant candidate evaluation, but total scores and broad candidate categories do not necessarily show how individual psychosocial concerns co-occur or which modifiable domains require further clinical attention. This study describes the development and internal evaluation of a clinical decision-support application that reorganizes Stanford Integrated Psychosocial Assessment for Transplantation (SIPAT) data into structured multidomain profiles. **Methods:** The application was developed using a retrospective single-center dataset of 496 adult lung transplant candidates. It integrates the SIPAT total score and candidate category with seven SIPAT-derived domain indicators, domain burden scores, cohort-referenced z-scores, graphical displays, and a ranked summary of domains for clinical review. All seven indicator targets were prespecified deterministic functions of SIPAT items or domain scores obtained during the same assessment. Random forest algorithms with sigmoid calibration were used to transform these targets into percentage-scaled display values; they were not trained using independently assessed clinical outcomes. Analyses included descriptive statistics, Spearman correlations, exploratory clustering, resampling-based cluster stability assessment, threshold sensitivity analyses, and subgroup analyses by age, sex, and primary pulmonary diagnosis. **Results:** Upper display-band classifications were identified for depression-related concerns in 32 candidates (6.5%), anxiety-related concerns in 11 (2.2%), nicotine-related concerns in 56 (11.3%), alcohol-related concerns in 22 (4.4%), illicit drug-use concerns in 11 (2.2%), social-support deficits in 40 (8.1%), and non-adherence-related concerns in 7 (1.4%). Exploratory clustering yielded a low-burden majority group (*n* = 432), a nicotine-dominant group (*n* = 53), and a small multidomain-elevation group (*n* = 11). The generated percentage-scaled indicators were positively associated with their conceptually corresponding SIPAT domains (Spearman’s ρ = 0.321–0.774) and with the total SIPAT score (ρ = 0.406–0.802; all *p* < 0.001). Sensitivity analyses showed that the smaller clusters were less stable under bootstrap resampling. These findings demonstrate internal alignment with the source instrument but do not constitute validation against independent clinical outcomes. **Conclusions:** The application provides an early-stage framework for organizing and visualizing SIPAT information and identifying domains that may warrant additional clinical assessment. Its outputs should be interpreted as SIPAT-derived decision-support indicators, not as independently validated probabilities of future clinical events. Prospective studies are required to evaluate usability, clinical impact, and associations with longitudinal outcomes.

## 1. Background

Psychosocial evaluation is a core component of transplant candidate assessment because successful transplantation requires more than biomedical eligibility. Candidates must understand the procedure and its consequences, participate in complex treatment, maintain behavioral adherence, manage psychological symptoms, avoid harmful substance use, and mobilize adequate practical and interpersonal support [1,2,3,4,5,6,7,8,9]. In the present study, psychosocial readiness is therefore defined as the candidate’s current capacity and available resources to meet these cognitive, emotional, behavioral, and social demands. It is understood as a multidimensional and potentially modifiable clinical state rather than a fixed personal characteristic, the absence of a psychiatric diagnosis, or a binary determination of transplant eligibility.

The Stanford Integrated Psychosocial Assessment for Transplantation (SIPAT) operationalizes this assessment across four broad domains: readiness and illness management, social support, psychological stability and psychopathology, and lifestyle and substance use [1,2]. The instrument provides item-level ratings, domain scores, a total score, and an overall candidate category. Its introduction has improved the standardization and documentation of psychosocial evaluation, while prospective research has demonstrated associations between SIPAT scores and selected medical and psychosocial outcomes [1,2].

Evidence specific to lung transplantation nevertheless indicates that the relationship between SIPAT scores, selection decisions, and subsequent outcomes is not uniform. In a study of 147 lung transplant candidates, Chernyak et al. found that the SIPAT total score and its social-support and psychopathology subscales were significantly associated with listing status [10]. Among 45 lung transplant recipients, Hinton-Froese et al. reported associations between pre-transplant SIPAT scores and the six-minute walk test, the number of readmissions, and the use of mental health services, but not organ rejection or mortality [11]. In a larger cohort of 914 candidates, Westall et al. found higher SIPAT scores among patients declined for transplantation; however, among listed patients, SIPAT scores were not associated with waitlist mortality or reduced post-transplant survival [12]. These findings support the value of structured psychosocial assessment while also showing that SIPAT should not be interpreted as a deterministic predictor of transplant outcomes.

A total score or candidate category summarizes overall psychosocial burden but does not by itself show how concerns co-occur within an individual candidate, how strongly each domain differs from the local reference cohort, or which areas should be reviewed first by the clinical team. Although this information remains available in the underlying SIPAT items, extracting and integrating it manually may be time-consuming. The relevant clinical gap is therefore not the absence of psychosocial information but rather the absence of a standardized method for reorganizing that information into a concise patient-level display.

Prehabilitation is a multimodal preoperative strategy intended to enhance patients’ preparedness and functional reserve before major surgery through physical, nutritional, metabolic, and psychological interventions [13,14]. Psychosocial prehabilitation is defined here as the assessment-linked use of interventions before transplantation to strengthen modifiable psychological, behavioral, and social resources. Depending on the candidate’s profile, these interventions may include psychoeducation, treatment of anxiety or depressive symptoms, motivational or relapse-prevention work, adherence planning, and clarification or strengthening of caregiver support. This approach differs from gatekeeping because identified concerns are treated primarily as targets for further assessment and intervention rather than automatically as grounds for exclusion. The European Society of Organ Transplantation includes psychological and psychosocial interventions within multimodal prehabilitation, although the available evidence remains heterogeneous and the certainty of the evidence for specific psychosocial interventions is generally low [14,15].

More broadly, advances in artificial intelligence and machine learning have expanded the use of computational approaches for synthesizing complex clinical data while emphasizing the importance of interpretability and integration with clinical workflows [16,17,18].

Clinical decision-support systems may facilitate this approach by organizing multiple data elements into standardized and interpretable displays [16,17,19,20]. In the present application, this means presenting the SIPAT total score and candidate category together with seven SIPAT-derived domain indicators, raw domain burden scores, cohort-referenced z-scores, graphical summaries, and a ranked list of domains for further clinical review. These outputs are intended to direct clinicians’ attention to potentially modifiable concerns. They are not intended to predict post-transplant outcomes, replace clinical judgment, or determine listing eligibility.

All seven indicator targets were constructed directly from information recorded during the same SIPAT assessment that provided the application inputs. The application therefore does not add new clinical observations to SIPAT and does not claim incremental, diagnostic, or prognostic validity beyond the source instrument. Its proposed added value is organizational: the simultaneous presentation of item-derived concerns, absolute domain burden, cohort-referenced values, multidomain configurations, and graphical summaries within a single interface. Accordingly, the present study is framed as an early-stage clinical decision-support prototype rather than a clinical prediction model study. Reporting was informed by contemporary guidance on artificial intelligence and clinical decision support while recognizing that conventional prediction model validation requires outcomes assessed independently of the model inputs [21,22,23].

The present developmental and internally evaluative study addressed four research questions: (1) What is the distribution of the seven SIPAT-derived domain indicators in the study cohort? (2) Do exploratory clustering methods identify recurring multidomain configurations within these indicators? (3) How closely are the generated indicators associated with their corresponding SIPAT domains and the total SIPAT score? and (4) What information does the application present at the individual-patient level beyond the total SIPAT score and candidate category? Because no independent longitudinal outcomes were included, the study did not test a confirmatory clinical prediction hypothesis. We expected positive associations between the generated indicators and both their conceptually corresponding SIPAT domains and the total SIPAT score. We also expected exploratory clustering to identify a low-burden majority together with smaller subgroups characterized by elevated domain-specific or multidomain concerns.

## 2. Materials and Methods

### 2.1. Study Design, Setting, and Participants

This retrospective developmental study used routinely collected clinical data from 496 adults undergoing their initial inpatient lung transplant evaluation at the Lung Transplantation Unit or the Clinic of Pulmonology, University Clinical Center in Gdańsk, Poland, between December 2021 and November 2025. Evaluations were conducted as part of the center’s standard transplant qualification pathway. Psychosocial assessment was performed during the routine multidisciplinary transplant evaluation and was not introduced specifically for the purposes of this study.

No prospective recruitment, advertising, or direct invitation of participants was undertaken. Recruitment for the present analysis consisted of the retrospective identification of consecutive clinical records from the standard inpatient transplant qualification pathway. Psychosocial evaluations were conducted by clinical psychologists experienced in transplant medicine and advanced pulmonary disease. Information was obtained through a semi-structured clinical interview and a review of medical documentation, supplemented, when clinically necessary, by consultation with the treating team.

Inclusion criteria were: (1) age of 18 years or older at the time of assessment; (2) evaluation for lung transplantation at the study center; (3) completion of a routine psychosocial assessment using the SIPAT; and (4) availability of sufficient item-level SIPAT data to generate the application outputs. Exclusion criteria were: (1) age below 18 years; (2) absence of a completed SIPAT assessment; and (3) item-level missingness that prevented the calculation of the required domain indicators. Candidates were not excluded on the basis of sex, underlying pulmonary diagnosis, listing decision, or subsequent receipt of transplantation.

The analytical dataset comprised 496 consecutive clinical records that met the stated eligibility criteria and contained sufficient SIPAT information to generate the application outputs. Because listing status and subsequent transplantation were not endpoints of this study, candidates were retained regardless of their subsequent listing decision or receipt of transplantation.

Age was unavailable for two candidates, whereas sex and primary pulmonary diagnosis were available for the entire analytical cohort. Participant characteristics were summarized using available-case denominators.

The study was conducted within the Polish healthcare and cultural context. Race and ethnicity were not routinely recorded in the source clinical dataset and could not be analyzed. The cohort should therefore be interpreted as a single-country clinical sample, and the cultural generalizability of the findings to transplant systems operating in other linguistic, cultural, or healthcare contexts remains to be established.

Study data were extracted between January and March 2026 and anonymized before analysis. No identifiable patient information was available in the analytical dataset. No research-specific assessment, intervention, or modification of clinical care was introduced.

### 2.2. Psychosocial Assessment

Psychosocial functioning was assessed using the SIPAT instrument, which evaluates multiple domains relevant to transplant candidacy. The instrument consists of item-level ratings grouped into four primary domains: readiness for transplantation, social support, psychological functioning, and substance use.

Each item is scored according to predefined criteria, reflecting the severity or presence of specific psychosocial factors. Domain scores are calculated by summing item-level values within each domain, and the total SIPAT score provides an overall index of psychosocial risk.

For analytical purposes, both item-level data and aggregated domain scores were used. Domain-level summaries included readiness, social support, psychological functioning, and substance use, as well as the total SIPAT score.

### 2.3. Definition of SIPAT-Derived Indicators and Computational Pipeline

A clinical decision-support research prototype was developed using a Python-based framework with an interactive web interface. The application accepts patient age and SIPAT item scores as inputs and produces four groups of outputs: (1) the original SIPAT total score and candidate category; (2) seven SIPAT-derived domain indicators covering depression-related concerns, anxiety-related concerns, nicotine-related concerns, alcohol-related concerns, illicit drug-use concerns, social-support deficits, and non-adherence-related concerns; (3) absolute SIPAT domain burden scores and cohort-referenced z-scores; and (4) graphical displays and a ranked summary of domains for further clinical review.

The seven model targets were deterministic operationalizations of the source SIPAT assessment. Depression-related concern was defined as SIPAT item IXa ≥ 2; anxiety-related concern as item IXb ≥ 2; nicotine-related concern as item XVIII ≥ 3; alcohol-related concern as item XV ≥ 2; illicit drug-related concern as item XVII ≥ 2; social-support deficit as an SIPAT Domain B score ≥ 6; and non-adherence-related concern as item IV = 4. All targets were available for all 496 candidates. They were recorded during the same pre-transplant assessment as the model inputs and were not based on independent psychiatric diagnoses, validated symptom scales, biological verification, pharmacy records, post-transplant adherence, or longitudinal clinical events. No separate adjudication or blinded outcome assessment was performed. Their provenance and interpretation are summarized in Supplementary Table S1.

Predictor inputs comprised age and 21 item-level SIPAT variables: items I–V, VI–VIII, IX, IXa, IXb, X, Xa, XI–XIII, and XIV–XVIII. All SIPAT item values were complete. Age was missing for two candidates and was replaced with the cohort median before model fitting. No algorithmic feature-selection procedure was applied.

Six first-stage random forest classifiers were fitted for the depression-, anxiety-, nicotine-, alcohol-, illicit drug-, and social-support targets. Each classifier used 500 trees, class-weight balancing, a minimum of three observations per terminal leaf, unrestricted tree depth, and a fixed random seed of 42. The remaining parameters retained their scikit-learn defaults. Hyperparameters were prespecified and were not optimized using data-driven tuning. Probability scaling used sigmoid calibration (Platt scaling), implemented with five-fold cross-validation. Isotonic regression was not used. The numerical output returned by predict_proba was multiplied by 100 and used as a percentage-scaled display indicator.

The non-adherence-related indicator was generated in a second stage using the six first-stage percentage-scaled indicators as inputs. The second-stage model used the same random forest and sigmoid-calibration parameters. Because the target-defining SIPAT items or domain scores were included among the model inputs, the algorithms learned computational representations of relationships already contained within SIPAT.

No independent training/test split was used. The models were developed and summarized in the same 496-candidate cohort. The five-fold procedure formed part of the sigmoid-calibration algorithm and should not be interpreted as independent internal or external validation. The resulting values are therefore display-scale transformations of SIPAT-derived information rather than calibrated probabilities of independently assessed clinical outcomes.

Conventional prediction-performance measures, including AUROC, precision–recall AUC, Brier score, calibration slope, and decision-curve net benefit, were not interpreted as measures of clinical validity. Against deterministically SIPAT-derived targets, such statistics would primarily quantify reconstruction of the source rules and could misleadingly suggest independent predictive performance [24,25]. Interpretation was therefore based on transparent target definitions, descriptive distributions, internal alignment with SIPAT scores, and exploratory profile analysis. Impurity-based feature importance was not retained in the revised analysis because of its known susceptibility to bias and because the explicit target definitions provide a more transparent account of indicator provenance [26].

**Figure 1:**
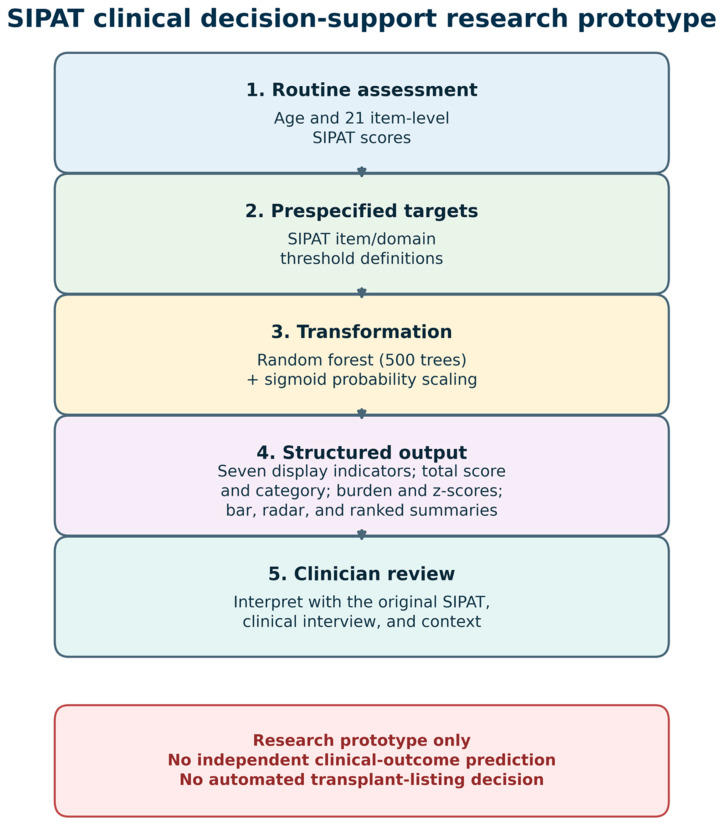
Conceptual workflow of the SIPAT clinical decision-support research prototype. Age and item-level SIPAT scores are processed using prespecified SIPAT-derived target definitions and random forest-based computational transformations. The application presents structured display indicators together with the original SIPAT score, candidate category, domain burden measures, and graphical summaries for clinician review. The prototype does not independently predict clinical outcomes or generate automated transplant-listing decisions.

For each candidate, the interface displays the numerical value and display band of each indicator, the original SIPAT results, bar-chart and radar-chart visualizations, cohort-referenced domain burden, and a ranked list of domains with the greatest relative elevation. The prototype does not generate an automated listing recommendation, and all outputs require interpretation in conjunction with the original SIPAT assessment and clinical interview. The computational workflow is summarized in Figure 1.

### 2.4. Risk Categorization

For interface-display purposes, the percentage-scaled indicators were grouped into three descriptive bands: lower (<20%), intermediate (20–49%), and upper (≥50%). These pragmatic bands were applied uniformly to facilitate visual organization within the research prototype. They were not derived from clinical outcomes, sensitivity/specificity analyses, decision curves, or expert consensus thresholds and have not been validated as diagnostic, prognostic, or treatment-triggering cutoffs. Accordingly, they describe relative positions on the application’s display scale rather than probabilities that future clinical events will occur. Sensitivity analyses examined the number of candidates exceeding alternative upper-band thresholds of 40%, 50%, and 60%.

### 2.5. Domain-Level Burden and Cohort Referencing

Absolute domain burden scores were calculated by summing the original SIPAT item scores assigned to each of the four SIPAT domains. These scores retain the original scale and provide a direct summary of the concerns documented during the clinical assessment.

For cohort-referenced presentation, each domain score was also standardized as z = (individual score − cohort mean)/cohort standard deviation. Positive values indicate greater burden than the mean observed in the study cohort, whereas negative values indicate lower burden. These z-scores are descriptive, center-specific reference values rather than external norms. They are used to identify domains that warrant comparative attention and do not independently establish the presence of a clinical disorder or unmet treatment need.

### 2.6. Statistical Analysis

Descriptive statistics were calculated for the seven percentage-scaled indicators and the original SIPAT item, domain, and total scores. The numbers and percentages of candidates in each descriptive display band were reported. Wilson 95% confidence intervals were calculated for the proportions classified in the upper display band. To distinguish deterministic source-rule positivity from upper display-band assignment, each deterministic source rule was cross-tabulated against its corresponding percentage-scaled application output dichotomized at ≥50%. True-positive, false-positive, true-negative, and false-negative counts were calculated with the deterministic SIPAT-derived rule treated as the reference. These counts were used solely to describe reconstruction concordance within the development cohort and were not interpreted as measures of diagnostic or prognostic performance against independently assessed clinical outcomes.

Associations between the seven continuous percentage-scaled indicators and the four SIPAT domain scores and total SIPAT score were assessed using Spearman’s rank correlation coefficients. For each indicator, the coefficient for the conceptually corresponding SIPAT domain and the coefficient for the total SIPAT score were reported. These analyses evaluated internal alignment with the source assessment and were not intended as validation against independent outcomes.

Exploratory k-means clustering was applied after standardization of all seven indicators using StandardScaler. The primary analysis specified three clusters, k-means++ initialization, 20 initial centroid configurations, a maximum of 300 iterations, the Lloyd algorithm, and a random seed of 42. Cluster labels were assigned post hoc from centroid patterns.

Sensitivity analyses compared solutions containing two to six clusters using silhouette coefficients, Calinski–Harabasz indices, Davies–Bouldin indices, cluster size, parsimony, and clinical interpretability. Stability of the three-cluster solution was evaluated across 500 repetitions of 80% subsampling without replacement and 500 bootstrap samples. Resampled solutions were compared with the primary solution using the adjusted Rand index and cluster-specific Jaccard coefficients.

Threshold sensitivity was examined by comparing the numbers of candidates exceeding upper-band thresholds of 40%, 50%, and 60%. Equity-oriented exploratory analyses examined associations between the continuous indicators and age using Spearman correlations, differences according to sex using Mann–Whitney U tests, and differences across 15 primary pulmonary diagnosis groups using Kruskal–Wallis tests. Holm correction was applied separately across the seven indicators within each family of subgroup tests. These analyses were exploratory and were not intended to establish the absence of bias or the fairness of the prototype.

All analyses were conducted using Python 3.12.7 (Python Software Foundation, Beaverton, OR, USA), with NumPy 2.4.3, pandas 3.0.1, SciPy 1.17.1, and scikit-learn 1.8.0. NumPy, pandas, SciPy, and scikit-learn are community-developed open-source projects fiscally sponsored by NumFOCUS, Inc. (Austin, TX, USA). A fixed random seed of 42 was used for the primary model-fitting and clustering procedures.

### 2.7. Prototype Status, Data Governance, and Equity Considerations

The application remained a research prototype throughout the study and was not deployed in clinical practice. Access to the prototype was restricted to its developer. The application was not used to inform transplant listing, allocation, or treatment decisions; its outputs were not entered into medical records; and the prototype did not retain patient-level results. The anonymized analytical dataset was stored on a password-protected computer.

Institutional permission was obtained from the University Clinical Center in Gdańsk to use anonymized routinely collected clinical data for research purposes. No application was submitted to an ethics committee or institutional review board; consequently, no formal exemption or waiver was issued.

Data on socioeconomic status, education, geographic distance from the transplant center, caregiver availability, and other detailed social determinants of health were not available. Equity-oriented analyses were therefore limited to age, sex, and primary pulmonary diagnosis. Before any future clinical implementation, separate prospective evaluation, institutional authorization, access control, model-version governance, output auditing, and procedures for monitoring unintended effects would be required. The prototype is intended to identify domains warranting further assessment or psychosocial support and must not be used as an automated gatekeeping or transplant-eligibility tool.

## 3. Results

### 3.1. Participant Characteristics

The analysis included 496 adult lung transplant candidates evaluated between December 2021 and November 2025 at a single tertiary transplant center in Gdańsk, Poland. Age was available for 494 candidates; the mean age was 57.20 years (SD = 10.70; range, 19–78). The cohort included 201 women (40.5%) and 295 men (59.5%). The most frequent diagnostic categories were COPD or emphysema (33.9%) and idiopathic or nonspecific interstitial lung disease (24.4%), followed by pulmonary hypertension (8.1%), connective tissue disease-related interstitial lung disease (7.5%), and hypersensitivity pneumonitis (6.2%). Complete demographic and clinical characteristics are presented in Table 1. Race and ethnicity were not routinely recorded in the source dataset; consequently, the cohort should be interpreted as a single-country clinical sample, and its generalizability to other cultural and healthcare settings remains to be established.

### 3.2. Distribution of SIPAT-Derived Display Indicators

The percentage-scaled indicators were concentrated predominantly in the lower display band. The most frequent upper-band classification concerned nicotine-related factors and was observed in 56 candidates (11.3%; 95% CI 8.8–14.4%). This was followed by social-support deficits in 40 candidates (8.1%; 95% CI 6.0–10.8%) and depression-related concerns in 32 candidates (6.5%; 95% CI 4.6–9.0%).

Upper-band alcohol-related concerns were identified in 22 candidates (4.4%; 95% CI 2.9–6.6%). Anxiety-related and illicit drug-related concerns were each identified in 11 candidates (2.2%; 95% CI 1.2–3.9%), while upper-band non-adherence-related concerns were identified in 7 candidates (1.4%; 95% CI 0.7–2.9%). Table 2 summarizes these classifications. They represent application-derived display bands and not the observed prevalence of psychiatric diagnoses, active substance use, substance relapse, or post-transplant non-adherence.

The frequencies presented in Table 2 refer to application outputs assigned to the upper display band at ≥50%, whereas the prevalence values presented in Supplementary Table S1 refer to positivity according to the deterministic SIPAT-derived source rules. The two counts were identical for the depression-, anxiety-, nicotine-, alcohol-, and illicit drug-related indicators, with complete reconstruction at the selected threshold. For the social-support indicator, 37 candidates were positive according to the deterministic source rule and 40 were assigned to the upper display band, corresponding to 37 true positives, 3 false positives, 456 true negatives, and no false negatives. For the non-adherence-related indicator, 10 candidates were source-rule positive and 7 were assigned to the upper display band, corresponding to 7 true positives, no false positives, 486 true negatives, and 3 false negatives. Complete reconstruction concordance results are provided in Supplementary Table S2. These comparisons quantify reconstruction of the deterministic SIPAT-derived rules within the development cohort and do not represent diagnostic performance, prognostic accuracy, or validation against independent clinical outcomes.

### 3.3. Exploratory Multidomain Clusters

Exploratory k-means analysis yielded three clusters. The labels were assigned after inspection of the cluster centroids and describe the dominant pattern within each cluster.

Cluster 1 included 432 candidates (87.1%) and was characterized by low values across all seven indicators, with no single dominant area of elevation. Cluster 2 included 53 candidates (10.7%) and was characterized by a pronounced elevation of the nicotine-related indicator (mean approximately 96%), while the remaining indicators were substantially lower. Cluster 3 included 11 candidates (2.2%) and showed concurrent elevations across several domains, including depression-related concerns (mean approximately 90%), anxiety-related concerns (approximately 82%), social-support deficits (approximately 67%), and non-adherence-related concerns (approximately 28%).

The study did not directly assess unmet treatment needs. Table 3 therefore reports potential areas for further clinical assessment suggested by each profile rather than confirmed intervention requirements. The clusters should be regarded as exploratory configurations within SIPAT-derived data and not as independently validated clinical phenotypes.

Comparison of two- to six-cluster solutions did not identify a single statistically dominant specification. Silhouette coefficients were 0.789 for two clusters, 0.691 for three clusters, 0.723 for four clusters, 0.735 for five clusters, and 0.786 for six clusters. The three-cluster solution was retained for descriptive reporting because it provided a parsimonious distinction between a low-burden majority, a nicotine-dominant subgroup, and a multidomain-elevation subgroup without producing numerous small profiles.

In the 80% subsampling analyses, agreement with the primary three-cluster solution was high (median adjusted Rand index = 0.967; interquartile range, 0.936–0.990). Bootstrap stability was lower and more variable (median adjusted Rand index = 0.726; interquartile range, 0.342–0.967). Median bootstrap Jaccard coefficients were 0.926 for the low-burden cluster, 0.729 for the nicotine-dominant cluster, and 0.833 for the multidomain-elevation cluster. These findings indicate that the large cluster was comparatively stable, whereas the smaller profiles require external replication.

**Figure 2:**
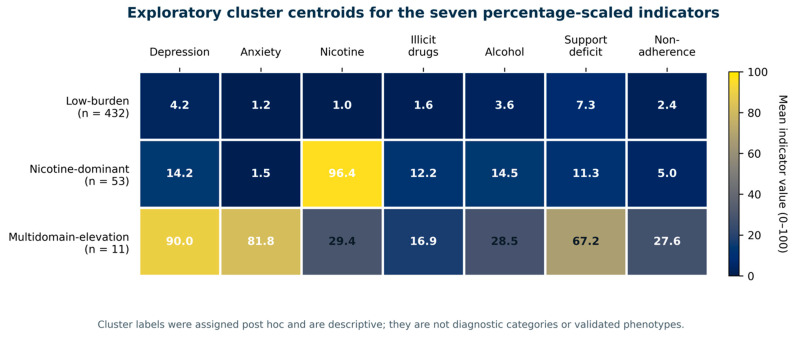
Exploratory cluster centroids for the seven percentage-scaled SIPAT-derived indicators. The three-cluster solution comprised a low-burden profile (*n* = 432), a nicotine-dominant profile (*n* = 53), and a multidomain-elevation profile (*n* = 11). Values represent mean indicator scores on a 0–100 scale. Cluster labels were assigned post hoc and should be interpreted as descriptive rather than diagnostic or independently validated psychosocial phenotypes.

Centroid patterns for the three clusters are presented in Figure 2.

### 3.4. Associations with SIPAT Domains

The seven continuous percentage-scaled SIPAT-derived indicators showed statistically significant associations with their conceptually corresponding SIPAT domains and with the total SIPAT score (Table 4). The depression- and anxiety-related indicators were associated with the psychological stability and psychopathology domain (Spearman’s ρ = 0.663 and 0.602, respectively; both *p* < 0.001).

The nicotine-, alcohol-, and illicit drug-related indicators were associated with the lifestyle and substance-use domain (ρ = 0.531, 0.478, and 0.462, respectively; all *p* < 0.001). The social-support deficit indicator was associated with the social-support domain (ρ = 0.774, *p* < 0.001), while the non-adherence-related indicator was associated with the readiness and illness-management domain (ρ = 0.321, *p* < 0.001). Associations with the total SIPAT score ranged from ρ = 0.406 to 0.802, with the largest coefficient observed for the anxiety-related indicator (ρ = 0.802, *p* < 0.001).

Because the generated indicators and reference scores were derived from the same SIPAT assessment, these associations demonstrate internal alignment with the source instrument rather than predictive validity against independently assessed clinical outcomes.

### 3.5. Threshold Sensitivity and Equity-Oriented Subgroup Analyses

Changing the upper-band threshold from 50% to 40% or 60% did not change the number of upper-band classifications for depression-, anxiety-, nicotine-, alcohol-, or illicit drug-related concerns. For social-support deficits, 44 candidates (8.9%) exceeded 40%, 40 (8.1%) exceeded 50%, and 39 (7.9%) exceeded 60%. For non-adherence-related concerns, the corresponding numbers were 9 (1.8%), 7 (1.4%), and 2 (0.4%). These analyses demonstrate numerical robustness for five indicators but do not provide clinical validation of any threshold.

No indicator differed significantly between women and men after Holm correction. Older age showed small negative associations with the anxiety-related indicator (ρ = −0.126; Holm-adjusted *p* = 0.025), the alcohol-related indicator (ρ = −0.286; Holm-adjusted *p* < 0.001), and the social-support deficit indicator (ρ = −0.163; Holm-adjusted *p* = 0.002). The remaining age associations were not statistically significant after correction.

Across the 15 primary pulmonary diagnosis categories, only the nicotine-related indicator differed significantly after Holm correction (adjusted *p* = 0.037). No corrected differences were observed for the other six indicators. Because several diagnostic groups were small, these findings should be interpreted as exploratory. The analyses do not establish equity or the absence of bias, particularly because socioeconomic and structural variables were unavailable and age was itself included among the model inputs.

### 3.6. Application Outputs

For each candidate, the application produced five complementary forms of information: (1) the original total SIPAT score; (2) the original SIPAT candidate category; (3) seven percentage-scaled SIPAT-derived domain indicators with descriptive lower, intermediate, or upper display bands; (4) absolute and cohort-referenced burden scores for the four SIPAT domains; and (5) bar-chart, radar-chart, and ranked-priority displays.

**Figure 3:**
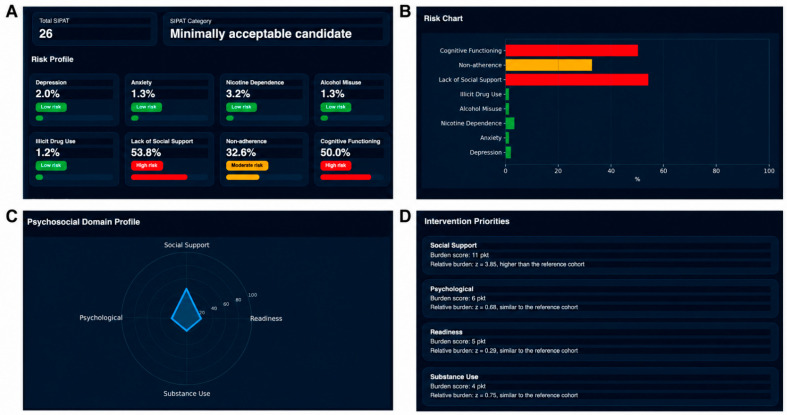
Representative interface outputs generated by the SIPAT clinical decision-support research prototype for a synthetic candidate. (**A**) Original SIPAT total score and candidate category, seven percentage-scaled SIPAT-derived indicators, and a separately displayed cognitive-functioning indicator; (**B**) horizontal risk-profile chart; (**C**) four-domain psychosocial burden profile; and (**D**) ranked domain summary intended to direct clinician attention to areas requiring further review. The percentage-scaled indicators are computational transformations of SIPAT data rather than validated probabilities of independently assessed clinical outcomes. The SIPAT candidate category reproduces the original instrument classification and does not constitute an automated transplant-listing recommendation.

The ranked display orders domains according to their relative elevation and is intended to indicate which parts of the original assessment should be reviewed first. It does not prescribe a specific intervention or make an automated transplant eligibility recommendation. Table 5 specifies the source, presentation, and intended interpretation of each output. Figure 3 presents an example of the research–prototype interface using synthetic input values. No identifiable patient data were used in the figure.

## 4. Discussion

This study describes the development and internal evaluation of a research prototype that reorganizes SIPAT item-level information into structured patient-level displays. Four principal findings emerged. First, upper display-band concerns were concentrated in relatively small subgroups, with nicotine-related factors being the most frequent. Second, exploratory clustering yielded a large low-burden group, a nicotine-dominant group, and a small multidomain-elevation group, although bootstrap analyses showed lower stability of the smaller profiles. Third, the generated indicators were associated with their conceptually corresponding SIPAT domains and the total SIPAT score, as expected from their construction. Fourth, exploratory subgroup analyses identified no corrected sex differences, while several small age- and diagnosis-related differences were observed. These findings characterize the internal behavior of the data transformation but do not establish clinical predictive validity, effectiveness, or fairness.

The results should be interpreted in the context of previous lung transplant research. Chernyak et al. found that the SIPAT total score and the social-support and psychopathology subscales were associated with listing status in 147 lung transplant candidates [10]. Hinton-Froese et al. subsequently reported that SIPAT scores were associated with six-minute walk performance, readmissions, and mental health service utilization during the first post-transplant year, but not rejection or mortality [11]. In a cohort of 914 candidates, Westall et al. found that higher SIPAT scores were associated with being declined for transplantation but did not predict waitlist mortality or post-transplant survival among listed patients [12]. Collectively, these studies indicate that SIPAT can standardize the identification of psychosocial concerns and may be associated with selected clinical processes or outcomes, but should not be treated as a deterministic prognostic instrument.

The exploratory clusters show how multiple SIPAT-derived concerns may co-occur within individual candidates. The nicotine-dominant cluster separated a specific substance-related pattern from the smaller multidomain-elevation cluster, in which psychological, social-support, and adherence-related concerns occurred together. Nevertheless, comparisons across alternative cluster numbers did not identify a uniquely optimal solution, and bootstrap analyses demonstrated variable reproducibility of the two smaller profiles. The clusters should therefore be interpreted as descriptive configurations generated within this cohort rather than stable or externally validated psychosocial phenotypes.

The associations with the corresponding SIPAT domains and total score were expected because both the targets and predictors originated from the same SIPAT assessment. The target definitions demonstrate that the generated indicators reorganize information already contained within the source instrument. They do not demonstrate the identification of new clinical constructs or the prediction of independently assessed depression, anxiety, substance-use relapse, non-adherence, rejection, hospitalization, or survival.

For the same reason, conventional discrimination, calibration, and decision-curve statistics calculated against the SIPAT-derived targets would primarily measure reconstruction of deterministic source rules. Such results could be numerically high while providing no evidence of clinical validity [21,22,24,25]. The present study therefore does not claim compliance as a validated clinical prediction model study. Its contribution lies in the prototype’s organization, visualization, and prioritization functions. Prospective outcomes assessed independently of SIPAT would be required before any probability or clinical utility claim could be made.

The intended clinical contribution of the application is organizational rather than prognostic. The three principal output types serve distinct purposes. Raw SIPAT domain sums retain the absolute burden recorded within the original instrument, while cohort-referenced z-scores show the candidate’s relative position within the local study cohort. In contrast, the random forest-derived percentage-scaled indicators reorganize prespecified SIPAT-derived concerns on a common display scale; they do not estimate probabilities of independently observed clinical events. Following completion of the SIPAT and the clinical interview, a clinician can use the interface to review the total score, candidate category, individual indicators, absolute domain burden, cohort-referenced values, and graphical summaries. The ranked summary can direct attention to domains requiring closer review, after which the clinician must return to the original SIPAT items and clinical information to determine whether further assessment or intervention is appropriate. The application is not intended to replace professional judgment or make automated listing decisions.

Compared with conventional clinical decision-support systems that integrate heterogeneous electronic health record variables to generate diagnostic, prognostic, or treatment recommendations, the present prototype operates entirely within a single established psychosocial assessment. This design improves traceability to the original SIPAT items and supports transparent clinical review, but it does not add independent clinical information or outcome-based prediction. Unlike externally validated diagnostic or prognostic decision-support tools, the present application has not undergone external validation, prospective implementation, or formal usability or clinical-impact evaluation. Its current role is therefore closer to an instrument-specific visualization and prioritization layer than to an autonomous prediction or recommendation system [16,19,20,23].

Recent literature further emphasizes the need for cautious and transparent evaluation of artificial intelligence-supported tools in transplantation. A 2024 systematic review of artificial intelligence models for heart and lung post-transplant outcomes reported promising discrimination but frequent high risk of bias and limited demographic representation [27]. A 2025 explainable artificial intelligence model for renal transplant readmission illustrates the use of SHAP and LIME to improve interpretability while also emphasizing the need for external validation [28]. More broadly, the FUTURE-AI consensus guideline identifies fairness, universality, traceability, usability, robustness, and explainability as core requirements for trustworthy healthcare artificial intelligence [29]. These principles support the present prototype’s traceability to the original SIPAT inputs and conservative presentation while also highlighting the external-validation, usability, robustness, and equity evaluations required before clinical implementation.

This use is consistent with a prehabilitation-oriented approach in which potentially modifiable concerns are linked to assessment and intervention rather than treated solely as exclusion criteria. The ESOT consensus statement recommends patient-tailored multimodal prehabilitation and identifies psychological and stress-management interventions as relevant components while emphasizing that the evidence base remains limited and heterogeneous [14]. In this context, the application may assist in matching the focus of further assessment to the candidate’s profile. The present study did not, however, examine whether clinicians used the outputs, whether the outputs changed clinical management, or whether profile-guided interventions improved patient outcomes.

The exploratory subgroup analyses do not establish that the prototype is equitable. The absence of corrected sex differences does not exclude bias, and the observed age- and diagnosis-related differences may partly reflect the structure of the SIPAT inputs and the inclusion of age as a model feature. Moreover, socioeconomic status, education, geographic access, caregiver availability, disability-related barriers, and other social determinants were unavailable. Social-support and adherence criteria may reflect structural disadvantage as well as individual behavior and should not be used automatically to restrict transplant access [30,31]. The prototype must therefore be positioned as a means of identifying candidates who may require additional resources, assessment, or prehabilitation rather than as a gatekeeping algorithm.

Cohort-referenced z-scores offer additional context by showing whether a candidate’s domain score is relatively elevated compared with other candidates evaluated at the same center. This reference is local and descriptive rather than normative. It may be influenced by referral pathways, center-specific selection practices, the clinical composition of the cohort, and the Polish cultural and healthcare context. Values should therefore not be transferred directly to other centers without local evaluation.

The available diagnostic categories captured broad clinical heterogeneity, including cystic fibrosis, alpha-1 antitrypsin deficiency within the COPD/emphysema group, and interstitial lung diseases. However, confirmed genetic variants, familial pulmonary fibrosis, telomere biology disorders, disease severity, oxygen or ventilatory support, and detailed transplant indications were not available. These clinical and genetic factors may influence transplant trajectories independently of psychosocial functioning and should be incorporated into future evaluations where available [32].

Several limitations should be considered. First, the retrospective single-center design limits generalizability and may introduce selection bias related to referral and completion of psychosocial evaluation. The study cohort was drawn from one Polish transplant system, and race, ethnicity, detailed cultural variables, socioeconomic indicators, education, geographic access, and caregiver availability were not recorded.

Second, the seven targets were deterministic functions of SIPAT items or domain scores and were not independent clinical outcomes. Because the target-defining information was included among the predictors, the modeling procedure was structurally circular. At the 50% display threshold, the social-support and non-adherence-related indicators did not perfectly reproduce source–target status, further demonstrating that upper display-band assignment is not interchangeable with deterministic source-rule positivity. The percentage-scaled outputs should therefore not be interpreted as probabilities of independently assessed disorders, behaviors, or post-transplant events. No independent test cohort, external validation, or prospective clinical outcome assessment was performed. The relatively small single-center cohort, the use of the same dataset for model development and descriptive evaluation, and the small numbers of positive source targets—particularly anxiety-related, illicit drug-related, and non-adherence-related concerns—create a risk of overfitting, model instability, and optimistic apparent performance.

Third, the models were fitted and summarized in the same development cohort. The five-fold sigmoid-calibration procedure did not constitute independent validation, and no hyperparameter tuning or formal comparison with simpler rule-based, penalized-regression, ordinal, or Bayesian approaches was undertaken. A transparent rule-based implementation may ultimately prove more appropriate for deterministic SIPAT-derived targets.

Fourth, the lower, intermediate, and upper display bands were pragmatic interface conventions and were not derived from external outcomes, clinical utility, intervention capacity, or expert consensus. Although sensitivity analyses demonstrated stable classification counts across alternative thresholds for five indicators, this does not establish clinical validity.

Fifth, the clustering was exploratory. Alternative values of k produced competing solutions, and bootstrap analyses showed variable reproducibility of the smaller clusters. The multidomain-elevation cluster included only 11 candidates, substantially limiting confidence in its stability and generalizability.

Sixth, SIPAT ratings were obtained during routine clinical assessment, and inter-rater reliability was not evaluated. The local cohort-referenced z-scores are center-specific and should not be treated as external norms. Clinical severity, oxygen and ventilatory support, detailed transplant indication, confirmed genetic conditions, and longitudinal transplant outcomes were unavailable.

Seventh, the subgroup analyses were restricted to age, sex, and diagnosis and cannot establish fairness across socioeconomic, cultural, disability-related, or structural dimensions. Finally, the prototype was not used clinically, and no formal usability testing, clinician-feedback study, prospective implementation assessment, comparison of clinical decisions with and without the application, or evaluation of patient outcomes was performed.

Future studies should first compare the current computational transformation with simpler transparent rule-based and shrinkage-based approaches. Subsequent work should use independent and multicenter cohorts, prospectively assessed psychological and behavioral outcomes, post-transplant adherence and clinical events, and prespecified external validation procedures. Research should also evaluate threshold selection, cluster reproducibility, inter-rater variability, usability, clinician interpretation, changes in clinical management, and potential unintended effects. Equity assessment should include socioeconomic position, education, caregiver availability, geographic access, disability-related barriers, cultural context, and relevant clinical or genetic subgroups. Only after such studies should the outputs be considered for use as clinically interpretable probabilities or treatment-triggering thresholds.

## 5. Conclusions

The application provides a structured method for organizing and visualizing multidomain information already contained within the SIPAT assessment. It identifies domain-specific and co-occurring patterns that may warrant further clinical review, but it does not generate independently validated probabilities of future outcomes or automated transplant recommendations. The findings support further prospective evaluation of the application as an early-stage clinical decision-support and psychosocial prehabilitation planning tool.

## Figures and Tables

**Table 1:** Sociodemographic and clinical characteristics of the study cohort (*n* = 496).

Characteristic	Category	*n*	Value
Age, years	Mean ± SD (range)	494	57.20 ± 10.70 (19–78)
Sex	Women	201	40.5%
	Men	295	59.5%
Primary pulmonary diagnosis	COPD/emphysema, including A1AT deficiency and asthma–COPD overlap	168	33.9%
	Idiopathic/nonspecific ILD, including IPF	121	24.4%
	Pulmonary arterial/chronic pulmonary hypertension	40	8.1%
	CTD-associated ILD	37	7.5%
	Hypersensitivity pneumonitis	31	6.2%
	Post-COVID chronic lung disease, ILD/mixed presentation	21	4.2%
	Other/mixed diagnoses, including cardiac and oncological conditions	17	3.4%
	Sarcoidosis	17	3.4%
	Post-transplant complications, previous heart–lung transplantation, or BOS	12	2.4%
	Non-CF bronchiectasis	10	2.0%
	Pneumoconiosis	8	1.6%
	Lymphangioleiomyomatosis	5	1.0%
	Asthma without clearly documented COPD or ILD	5	1.0%
	Cystic fibrosis/CF-related bronchiectasis	3	0.6%
	Other rare ILD, including LIP	1	0.2%

***Note:*** Age was available for 494 of 496 candidates; sex and primary pulmonary diagnosis were available for the entire cohort. Percentages were calculated using *n* = 496 and may not sum to exactly 100% because of rounding. A1AT, alpha-1 antitrypsin deficiency; BOS, bronchiolitis obliterans syndrome; CF, cystic fibrosis; COPD, chronic obstructive pulmonary disease; CTD-ILD, connective tissue disease-associated interstitial lung disease; ILD, interstitial lung disease; IPF, idiopathic pulmonary fibrosis; LIP, lymphocytic interstitial pneumonia.

**Table 2:** Frequency of upper display-band classifications for SIPAT-derived indicators.

Indicator	*n*	%	95% CI
Depression-related concerns	32	6.5	4.6–9.0
Anxiety-related concerns	11	2.2	1.2–3.9
Nicotine-related concerns	56	11.3	8.8–14.4
Alcohol-related concerns	22	4.4	2.9–6.6
Illicit drug-use concerns	11	2.2	1.2–3.9
Social-support deficits	40	8.1	6.0–10.8
Non-adherence-related concerns	7	1.4	0.7–2.9

***Note:*** The upper display band was defined as an application output ≥50%. Display bands are descriptive and were not validated against independent diagnoses or longitudinal clinical events. Confidence intervals were calculated using the Wilson method.

**Table 3:** Characteristics and potential clinical interpretation of the exploratory clusters.

Cluster	*n* (%)	Dominant Profile	Selected Mean Indicators	Potential Areas for Clinical Review
Low-burden profile	432 (87.1)	Low values across all indicators	No dominant elevation	Routine psychosocial review and monitoring
Nicotine-dominant profile	53 (10.7)	Marked nicotine-related elevation	Nicotine-related indicator ≈ 96%	Current nicotine status, abstinence verification, relapse-prevention planning
Multidomain-elevation profile	11 (2.2)	Concurrent psychological, social support, and adherence-related elevations	Depression ≈ 90%; anxiety ≈ 82%; social-support deficit ≈ 67%; non-adherence-related indicator ≈ 28%	Comprehensive psychological assessment, treatment planning, caregiver/support review, and individualized adherence planning

***Note:*** Cluster labels were assigned post hoc from centroid patterns and are descriptive rather than diagnostic. Potential areas for review were inferred from the profile and were not measured as confirmed unmet needs.

**Table 4:** Associations of SIPAT-derived indicators with their conceptually corresponding SIPAT domains and the total SIPAT score.

Generated Risk Estimate	Corresponding SIPAT Domain	ρ with Corresponding Domain	ρ with Total SIPAT Score
Depression-related	Psychological stability and psychopathology	0.663	0.779
Anxiety-related	Psychological stability and psychopathology	0.602	0.802
Nicotine-related	Lifestyle and substance use	0.531	0.571
Alcohol-related	Lifestyle and substance use	0.478	0.527
Illicit drug-related	Lifestyle and substance use	0.462	0.406
Social-support deficit	Social support	0.774	0.787
Non-adherence-related	Readiness and illness management	0.321	0.483

***Note:*** Values are Spearman’s rank correlation coefficients. All analyses included 496 candidates, and all reported coefficients were statistically significant at *p* < 0.001. The generated indicators are continuous, percentage-scaled transformations of SIPAT item-level data and are therefore expected to remain associated with both their conceptually corresponding SIPAT domains and the total SIPAT score.

**Table 5:** Information generated by the clinical decision-support application.

Output	Source/Calculation	Presentation	Intended Interpretation
Total SIPAT score	Sum of original SIPAT items	Numerical score	Overall psychosocial burden according to SIPAT
Candidate category	Original SIPAT classification	Categorical label	Conventional overall SIPAT interpretation
Seven domain indicators	Random-forest transformation of item-level SIPAT data	Percentage-scaled value and descriptive level	Relative intensity of an SIPAT-derived concern; not a validated event probability
Absolute domain burden	Sum of items within each SIPAT domain	Numerical domain score	Direct domain-specific burden
Cohort-referenced burden	Domain z-score relative to the study cohort	Numerical value, bar chart, and radar chart	Relative position within the local cohort; not an external norm
Ranked domain summary	Ordering of domains by relative elevation	Prioritized list	Domains to review first during clinical interpretation; not an automated treatment recommendation

## Data Availability

The clinical dataset cannot be deposited in a public repository because of patient confidentiality and institutional data-protection requirements. Access to appropriately de-identified data may be considered following a reasonable request to the corresponding author, institutional authorization, and the execution of an appropriate data-sharing agreement.

## Supplementary Materials

The following supporting information can be downloaded at: https://www.mdpi.com/article/10.3390/jcm15155899/s1, Table S1: Operational definitions and provenance of the SIPAT-derived indicator targets; Table S2: Reconstruction concordance between deterministic SIPAT-derived source rules and application upper display-band classifications (*n* = 496).
