# Supplementary material for: From Assessment to Action: A Research Prototype for SIPAT-Based Multidomain Psychosocial Visualization and Prioritization in Lung Transplant Candidates
Source: J Clin Med. 2026 Jul 28;15(15):5899. doi: 10.3390/jcm15155899 (PMC13466366; doi:10.3390/jcm15155899)
Supplement: Supplementary file 1 [file jcm-15-05899-s001.zip › jcm-4317946-supplementary.pdf]

Supplementary Table S1

**Table S1.** Operational definitions and provenance of the SIPAT-derived indicator targets.

| Indicator | Source definition | Positive, n (%) | Timing and independence | Missingness | Adjudication/blinding | Intended meaning |
|---|---|---|---|---|---|---|
| Depression-related | SIPAT item IXa ≥2 | 32 (6.5%) | Same pre-transplant SIPAT; not independent | 0 | None; no blinded assessment | Threshold-coded depressive concern; not a diagnosis |
| Anxiety-related | SIPAT item IXb ≥2 | 11 (2.2%) | Same pre-transplant SIPAT; not independent | 0 | None; no blinded assessment | Threshold-coded anxiety concern; not a diagnosis |
| Nicotine-related | SIPAT item XVIII ≥3 | 56 (11.3%) | Same pre-transplant SIPAT; not independent | 0 | None; no biological verification | Elevated nicotine-related concern; not confirmed current use or relapse |
| Alcohol-related | SIPAT item XV ≥2 | 22 (4.4%) | Same pre-transplant SIPAT; not independent | 0 | None; no independent diagnostic assessment | Elevated alcohol-related concern; not an alcohol-use diagnosis |
| Illicit drug-related | SIPAT item XVII ≥2 | 11 (2.2%) | Same pre-transplant SIPAT; not independent | 0 | None; no toxicological verification | Elevated drug-related concern; not confirmed current use or relapse |
| Social-support deficit | SIPAT Domain B score ≥6 | 37 (7.5%) | Same pre-transplant SIPAT; not independent | 0 | None; no separate adjudication | Elevated SIPAT social-support burden; not a confirmed unmet need |
| Non-adherence-related | SIPAT item IV = 4 | 10 (2.0%) | Same pre-transplant | 0 | None; no longitudinal | Severe SIPAT-recorded |

| | | | | | | |
|---|---|---|---|---|---|---|
| | | | SIPAT; not independent | | adherence assessment | adherence concern; not observed post-transplant non-adherence |

Supplementary Table S2

**Table S2.** Reconstruction concordance between deterministic SIPAT-derived source rules and application upper display-band classifications (*n* = 496).

| Indicator | Source-rule positive, n (%) | Upper display band, n (%) | TP | FP | TN | FN |
|---|---|---|---|---|---|---|
| Depression-related | 32 (6.5%) | 32 (6.5%) | 32 | 0 | 464 | 0 |
| Anxiety-related | 11 (2.2%) | 11 (2.2%) | 11 | 0 | 485 | 0 |
| Nicotine-related | 56 (11.3%) | 56 (11.3%) | 56 | 0 | 440 | 0 |
| Alcohol-related | 22 (4.4%) | 22 (4.4%) | 22 | 0 | 474 | 0 |
| Illicit drug-related | 11 (2.2%) | 11 (2.2%) | 11 | 0 | 485 | 0 |
| Social-support deficit | 37 (7.5%) | 40 (8.1%) | 37 | 3 | 456 | 0 |
| Non-adherence-related | 10 (2.0%) | 7 (1.4%) | 7 | 0 | 486 | 3 |

*Note:* Source-rule positivity was determined using the deterministic SIPAT-derived definitions presented in Supplementary Table S1. The upper display band was defined as a percentage-scaled application output ≥50%. TP, true positive; FP, false positive; TN, true negative; FN, false negative, with the deterministic source rule treated as the reference. These values describe reconstruction concordance within the same development cohort and must not be interpreted as measures of clinical diagnostic performance, prognostic accuracy, or validation against independently assessed outcomes.
